# Pneumomediastinum after Third Molar Extraction: Case Report, Physiopathology, and Literature Review

**DOI:** 10.1155/2023/4562710

**Published:** 2023-08-03

**Authors:** Mélissa Peters, Firas Shall, Laurence Evrard

**Affiliations:** Erasmus Hospital, Lennik Road, 900 Brussels, Belgium

## Abstract

Wisdom tooth extraction is a common procedure in dentistry and stomatology. Subcutaneous emphysema is a rare postoperative complication and commonly remains localized. However, it may spread to the mediastinum, endangering the life of the patient. This present paper presents a case study of pneumomediastinum after wisdom tooth extraction without the use of a compressed air turbine and reviews the cases of subcutaneous emphysema after third molar extraction published in the literature since 2010. The aim of this work is to inform preventive measures, pathophysiological processes, and management related to this complication.

## 1. Introduction

Wisdom tooth extraction is a common procedure in dentistry and stomatology. The most common postoperative complications are pain, infection, bleeding, trismus, adjacent tissue injury, inferior alveolar and lingual nerve paresthesia, and negative reactions to anesthesia. Maxillary or mandibular fractures and subcutaneous emphysema are rare [1–4]. Emphysema commonly remains localized at the cervicofacial level; however, it may spread to the mediastinum, thereby endangering the life of the patient [1–4].

The first described case of subcutaneous emphysema after extraction occurred in 1870 and was published in 1900. It involved a patient who developed facial swelling while playing with the bugle after premolar extraction [5]. From 1960 to 2008, two literature reviews reported 106 cases of subcutaneous emphysema after various dental procedures, including restorative dentistry, extraction, and endodontic treatments as well as periodontal, orthognathic, and laser surgery [6, 7]. Most of these cases were attributable to the use of compressed air turbines [6, 7], but other causes identified were (during the procedure) the use of air syringes, spreading the mucoperiosteal flap too wide with retractors, and irrigation with hydrogen peroxide, as well as (after the procedure) an increase of pressure caused by the patient sneezing, inflating a balloon, or blowing the nose [6, 7].

Although rare, this complication can be life-threatening. Therefore, it is important to recognize it—as well as its possible complications—in order to adopt the best therapeutic management strategies. Accordingly, we herein report a case of subcutaneous emphysema complicated by pneumomediastinum after wisdom tooth extraction despite no high-pressure air turbine being used. We also review similar cases published in the literature since 2010 with the hope of collating preventive, diagnostic, and therapeutic data as well as relevant pathophysiological processes.

## 2. Case Presentation

A 26-year-old patient presented to the emergency department because he had experienced crepitus in the face and neck for the preceding 24 hours. The patient also reported mild chest pain upon deep inspiration and throat pain. His four wisdom teeth (18–38–48 impacted, 28 on arch) had been extracted under general anesthesia, without incident and without the use of an air turbine, 48 hours before presentation. The patient was a smoker (three cigarettes per day) and had no other relevant medical history. In terms of postoperative care, the patient had been recommended to use pain killers (ibuprofen [600 mg three times per day] and paracetamol [1 g four times per day]); antibiotic therapy with amoxicillin (1 g two times per day); and chlorhexidine mouthwash (three times per day).

Upon physical examination, his parameters were found to be within normal limits (temperature, 37.1°C; blood pressure, 120/70 mmHg; heart rate, 60 bpm; saturated O_2_, 100% ambient air), and his cardiopulmonary auscultation results were unremarkable. Subcutaneous crepitations were observed bilaterally in the frontal, retroauricular, temporal, jugal, and subclavicular areas. Palpation was not painful, and there was no swelling, redness, or heat. Submucosal crepitations were observed at the site of extraction of tooth 38. Otherwise, there was no dehiscence or signs of infection.

**Figure 1:**
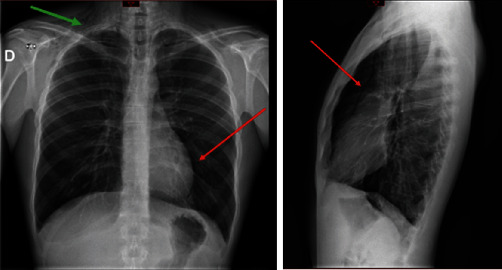
Chest radiograph of the face and profile. Supraclavicular subcutaneous emphysema (green arrow) and pneumomediastinum (red arrows; retrosternal radiolucency following the contour of the heart and aorta) are observed.

**Figure 2:**
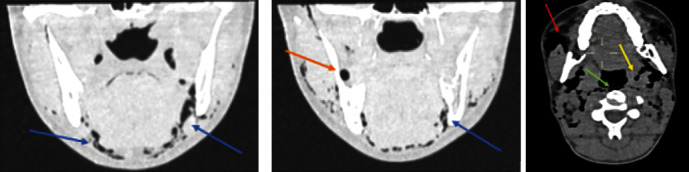
Cervicofacial computed tomography scan of the frontal and axial sections. Air is present in the extraction site of 48 (orange arrow) and in the submandibular (blue arrows), retropharyngeal (green arrow), parapharyngeal (yellow arrow), and masticatory spaces (red arrow).

**Figure 3:**
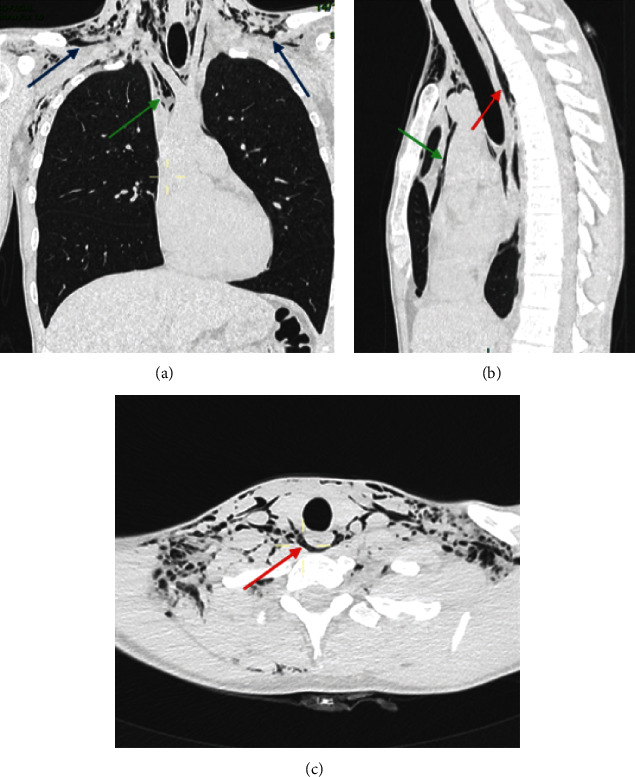
Chest computed tomography scan of the frontal, sagittal, and axial sections. Air is present in the supraclavicular soft tissue (blue arrows), mediastinum (green arrows), and retropharyngeal space (red arrows). (a) Chest computed tomography scan, frontal section. (b) Chest computed tomography scan, sagittal section. (c) Chest computed tomography scan, axial section.

Chest radiography (front and side) revealed subcutaneous supraclavicular soft tissue emphysema and pneumomediastinum (Figure 1). A cervicothoracic computed tomography (CT) scan without contrast injection confirmed parietal pneumatosis of the masticatory, submandibular, parapharyngeal, and retropharyngeal spaces (Figure 2) of the supraclavicular soft tissues with pneumomediastinum but without pneumothorax or pneumoperitoneum. There was no pericardial or pleural effusion or visible mediastinal collection. No tracheobronchial tree lesions were detected (Figure 3). An ecchymosis of the posterior wall of the nasopharynx was visualized using ear, nose, and throat fibroscopy without mucosal breach. Blood test results were unremarkable, and no inflammatory syndrome was observed.

The patient reported that he had not smoked, sneezed, coughed, or exerted pressure (e.g., inflated a balloon or played a wind instrument) since the intervention. The patient only reported becoming angry and quarrelling with his partner, and that he may have performed a Valsalva maneuver during the argument.

**Figure 4:**
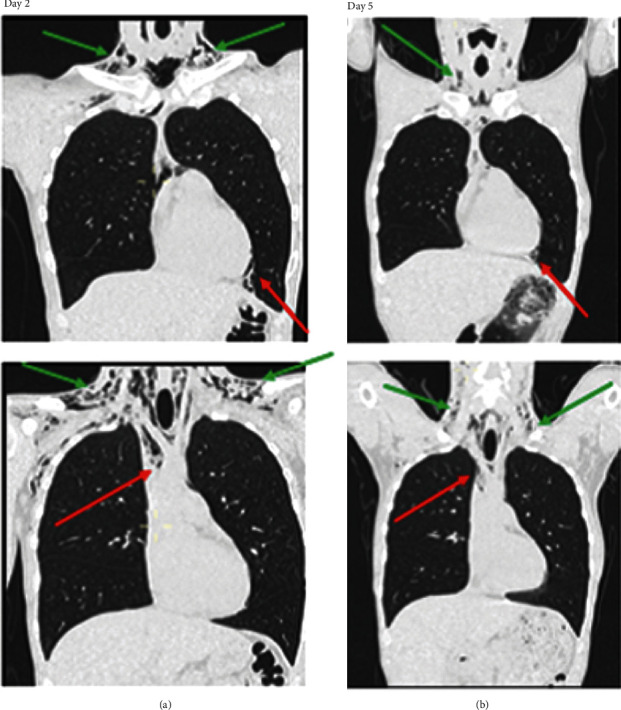
Cervicothoracic frontal slices obtained during the computed tomography scan. Subcutaneous supraclavicular soft tissue emphysema (green arrow) and pneumomediastinum (red arrow) from 2nd to the 5th day after extraction are reduced. (a) Cervicothoracic tomography scan, frontal slices, 2nd day after wisdom tooth extraction. (b) Cervicothoracic tomography scan, frontal slices, 5nd day after wisdom tooth extraction.

The patient was admitted to our department, intravenous antibiotic prophylaxis was initiated (amoxicillin–clavulanic acid, 2 g three times per day), and his parameters were monitored. He was strictly instructed to avoid blowing his nose and applying intraoral pressure. The patient remained stable. A follow-up CT scan and physical examination after 2 days of hospitalization showed a clear decrease in emphysema and pneumomediastinum (Figure 4). Therefore, he was able to return home. He was examined again 12 days after the extraction. At that time, the complete disappearance of crepitations was observed upon physical examination.

## 3. Discussion

Subcutaneous emphysema is a rare complication of wisdom tooth extraction and can lead to life-threatening complications [1–4]. A PubMed search of “(extraction) OR dental procedure) AND emphysema” yielded 26 additional cases of emphysema after wisdom tooth extraction that were published between January 2010 and April 2020. Tables 1, 2, and 3 provide details regarding the sex and age of the patients, extraction procedure, suspected cause, distribution of emphysema, treatment, and complications that occurred.

Subcutaneous emphysema is mainly attributable to iatrogenic, traumatic, infectious, and spontaneous causes (Table 4) [1, 3, 8–12]. After wisdom tooth extraction, an open cavity and air can be introduced under pressure, which can lead to subcutaneous emphysema formation [1, 3, 4]. This air often comes from the compressed air turbine used during surgery. However, it can also be introduced by an increase in pressure caused by the patient themself upon blowing the nose, sneezing, coughing, performing a Valsalva maneuver, vomiting, using mouthwash too vigorously, drinking through a straw, inflating a balloon, playing a wind instrument, using a continuous positive airway pressure machine, traveling by airplane after prolonged surgery, or by unknown means [2–4]. The cause of subcutaneous emphysema in this case report was uncertain because the extraction was performed with a conventional handpiece and without air injection. Therefore, the introduction of air may have occurred as a result of excessive coughing after extubation, using mouthwash too vigorously, smoking, or any other action that increased intraoral pressure but was not reported by the patient.

**Figure 5:**
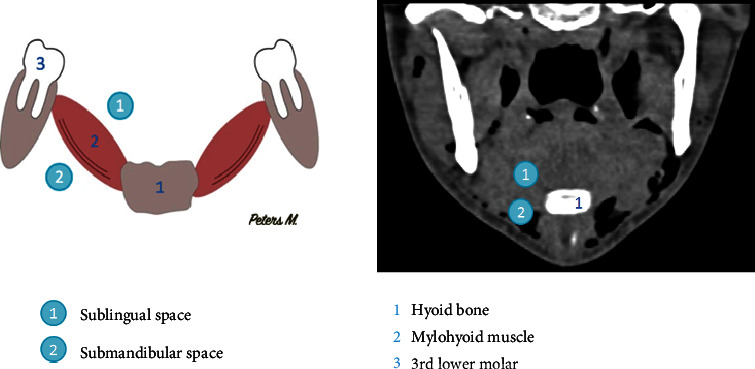
Schematic and computed tomography scan of the frontal section through the lower third molar of our patient. The roots of the lower molars are in contact with the submandibular space and separated from the sublingual space by the mylohyoid muscle.

**Figure 6:**
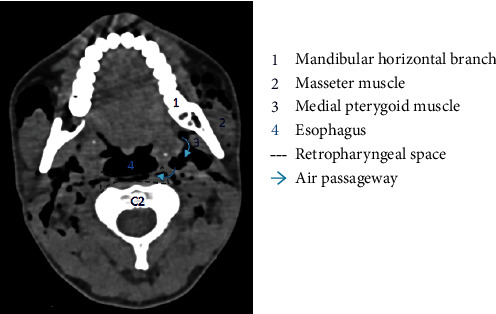
Computed tomography scan of the axial section through C2 of our patient. Air diffused from the submandibular space to the parapharyngeal space, and then to the retropharyngeal space.

**Figure 7:**
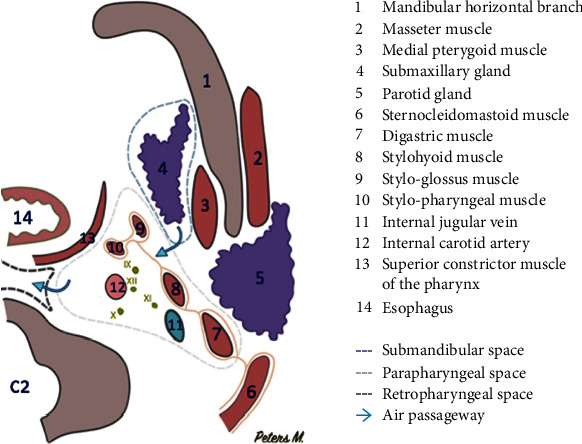
Schematic of the axial section through C2. Air can diffuse from the submandibular space to the parapharyngeal space, and then to the retropharyngeal space. IX: glossopharyngeal nerve; X: vagus nerve; XI: accessory nerve; XII: hypoglossal nerve.

**Figure 8:**
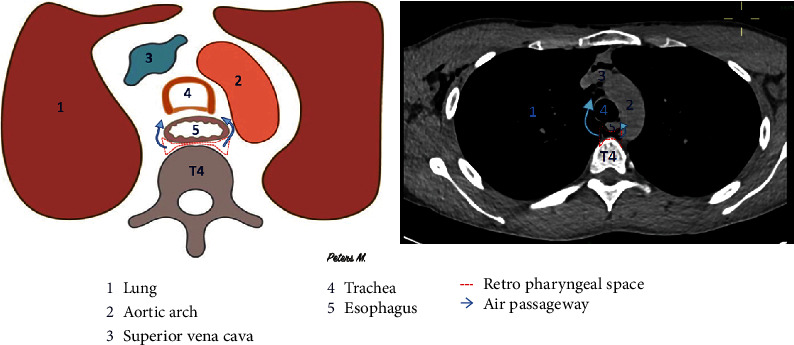
Schematic and computed tomography scan of the axial section through T4 of our patient. Air diffused from the retropharyngeal space to the mediastinum.

Regarding the physiopathology of subcutaneous emphysema, different superficial and deep fascias delimit spaces in the cervicofacial region. These spaces will communicate with each other, allowing the propagation of air [10]. At the mandibular level, the roots of the third molars are in contact with the submandibular space [11, 13, 14] and separated from the sublingual space by the mylohyoid muscle. We observed the presence of air in the submaxillary space in our patient (Figure 5). This air can progress from the submaxillary space to the parapharyngeal space, and then to the retropharyngeal space (Figures 6 and 7). This occurred in our patient. The retropharyngeal space communicates with the mediastinum [2, 10–12, 15–17], thus allowing air to continue to spread and cause pneumomediastinum, which was also observed in the present report (Figure 8). The incidence of air distribution in the 26 cases reported in the literature is presented in Table 3, with pneumomediastinum recorded in 20 patients (77% of cases).

Subcutaneous emphysema manifests directly a few hours or days after extraction, depending on the cause. Certain functions such as chewing, swallowing, and phonation can draw in air that had initially remained localized, which would explain why emphysema can spread long after the procedure has ended [2]. Our patient noticed it the day after extraction, but the cause was not clearly identified. Accordingly, the time at which the air was introduced is unknown.

Most patients will notice the presence of a swelling with subcutaneous crepitations; some may report neck pain, facial pain, chest pain, dyspnea, dysphagia, odynophagia, vocal hoarseness, or visual disturbance [8, 10, 18]. These different manifestations were observed in the 26 cases reported in the literature and are shown in Table 5. The subject in the present case had crepitations, throat pain, and chest pain as main symptoms, but no swelling.

The diagnosis is suspected from clinical history and clinical findings like swelling and subcutaneous crepitations, and it can be confirmed by chest radiology, which allows visualization of the air accumulation as radiolucent areas. However, one-third of the pneumomediastinum can be missed using this imaging method [23]. Therefore, a CT scan with contrast injection should be performed to determine the extent of emphysema and infectious complications [2, 9, 21]. Our patient underwent X-ray and CT examinations to diagnose emphysema and the presence of air in the mediastinum. However, a CT scan with contrast injection would have been more appropriate.

Diagnosis of subcutaneous emphysema is important because, if unnoticed, it can lead to various complications, such as pressure on the orbit, which can cause blindness [9, 13], compression of the upper airway that sometimes requires intubation of the patient [1, 3, 14], and nerve compression (such as that of the recurrent laryngeal nerve) that causes vocal cord paresis [1]. Furthermore, subcutaneous emphysema may extend to the thorax, causing pneumopericardium [19, 20] or pneumomediastinum [1, 3, 8, 10–12, 14–17, 19–28]. Pressurized air can rupture the mediastinal pleura and cause pneumothorax [11, 19, 26]. Air can also reach the spinal level, thereby causing pneumorrhagia with possible compression of the spinal canal [12]. Air can also pass to the bloodstream and form emboli [10, 19]. Infectious complications can also occur as a result of the diffusion of germs from the oral cavity or non-sterile irrigation into various spaces [9–15], thus leading to necrotizing fasciitis [14, 17] or mediastinitis [1, 4, 9, 10]. These are serious complications that are potentially life-threatening. Accordingly, clinical findings and the extent of emphysema are important to specify. In our patient, emphysema spread to the thorax and caused pneumomediastinum and the air remained localized in the mediastinum, without infection or other complications.

When evaluating a case of swelling after wisdom tooth extraction, it is necessary to make a differential diagnosis of an allergic reaction, hematoma, cellulitis, or subcutaneous emphysema because crepitations rapidly present in the case of subcutaneous emphysema and indicate the correct diagnosis [10, 15, 22]. Patient history will provide information about any previous dental extraction, the protocol used during the extraction, and whether there were increases in intraoral pressure after extraction [2, 3, 11]. The biology of emphysema is often normal. However, an increase in infectious parameters sometimes occurs [21].

Regarding therapeutic management, antibiotic prophylaxis should be initiated to avoid any infectious complications [10–12]. Amoxicillin–clavulanic acid seems to be the most frequently used antibiotic treatment [17]. Some studies have suggested oxygen therapy to eliminate accumulated air more rapidly [10–12, 19, 24], whereas others have advised performing skin or chest drainage [1, 8, 13]. However, other reports have indicated that this may instead create a new entry point for air, worsening the situation [9, 24]. In some cases, tracheostomy is necessary to protect the upper airway [3, 10]. The patient should be instructed not to apply intraoral pressure [12, 24]. As our patient's emphysema spread to the mediastinum, we preferred to hospitalize the patient to monitor his parameters and start intravenous antibiotic prophylaxis. Our patient did not need further treatment.

Subcutaneous emphysema usually remains localized to the cervicofacial area and will not require further treatment [11]. However, as we have observed, complications are possible and may be life-threatening [10, 14]. Numerous studies have shown that partial resorption occurs on day 4 or 5, and that complete resorption occurs on day 9 or 10 [1–4, 8–10, 12–18, 20–22, 24–28], which occurred in the subject of the current report. The evolution can be monitored by performing X-ray examinations or CT examinations. However, CT examinations more precisely visualize the extent of emphysema [24]. There are no recommendations regarding time frame for the new imagery. For our patient, we evaluated evolution with CT when, clinically, we observed fewer crepitations.

Regarding prevention, various authors have advised limiting the use of compressed air turbines and air injection as much as possible, limiting the detachment of mucoperiosteal flaps, limiting muscular disinsertion (especially at the lingual level), and sectioning the tooth before detachment [11, 13, 18]. It is important to extend the postoperative instructions and advise patients not to smoke, cough, or sneeze with the mouth open, not to play a wind instrument, not to inflate a balloon, not to perform a Valsalva maneuver, not to travel by plane, not to perform intense exercise, and to use mouthwash effectively but not too vigorously [1–4, 10, 12, 15, 16, 18, 21, 27]. In this case, the introduction of air may have occurred as a result of excessive coughing after extubation, using mouthwash too vigorously, smoking, Valsalva maneuver, or any other action that increased intraoral pressure but was not reported by the patient.

## 4. Conclusions

We have presented a case of pneumomediastinum after third molar extraction without the use of a compressed air turbine. The cause was not identified, but would probably be due to an increase in intraoral pressure with unknown etiology. The patient was hospitalized, treated with antibiotic prophylaxis, and evolved favorably.

We reviewed the relevant literatures available on this matter, reported since 2010. Although it remains rare, subcutaneous emphysema can extend between the different masticatory, submaxillary, parapharyngeal, and retropharyngeal spaces to the mediastinum and cause several complications. To avoid this situation, several measures can be performed during third molar extraction, such as limiting the use of dental handpieces (such as compressed air turbines or accelerator dental handpieces) during dental sectioning and opting for the use of surgical handpiece with sterile irrigation and no spray air. Limiting the mucoperiosteal detachment and providing the patients with the usual postoperative recommendations to avoid any increase in pressure likely to diffuse air into the deep spaces are also highly recommended. When a patient develops subcutaneous emphysema, the diagnosis should be based on clinical findings supplemented by chest X-ray and CT scan examination findings. The patient is usually hospitalized for monitoring and management when complications occur with antibiotic prophylaxis to avoid any infectious complications.

## Figures and Tables

**Table 1:** Characteristics and complications of 26 cases of subcutaneous emphysema after wisdom tooth extraction reported from January 2010 to April 2020.

First author (reference number)/year	Sex/age (years)	No. of teeth extracted	Possible causes	Complications
Kaliszewski [22]/2020	F/21	18	NS	PM
North [1]/2019	M/17	18	CAT	PM + cervical edema
Fehrle [25]/2019	M/32	48	Irrigation	PM
Paschos [26]/2019	F/17	38	CAT	PM + PT
Tay [12]/2018	M/18	48	Blowing nose	PM + PT + PR
Zaheer [23]/2018	F/55	48	CAT	PM
Jeong [24]/2018	F/27	28	NS	—
	F/22	18 + 48	NS	PM
Thompson [8]/2017	M/50	38	CAT	PM
Ocakcioglu [11]/2016	M/23	48	CAT	PM + PT
Picard [16]/2015	M/26	48	CAT, vigorously using mouthwash	PM
Tomasetti [4]/2015	M/30	38 + 48	Valsalva maneuver	—
Aslaner [10]/2015	F/33	48	CAT	PM
Johannesma [20]/2014	F/33	48	CAT	PM + PP
Fleischman [13]/2014	F/15	28	NS	—
Baisi [27]/2014	M/16	48	Swimming	PM
Lim [9]/2014	F/30	38	CAT	—
Kün-Darbois [17]/2014	F/41	38	CAT	PM
Olate [2]/2013	F/23	48	CAT	—
Pilar [15]/2012	M/30	48	CAT	PM
Chen [19]/2012	F/25	28	CAT	PM + PP
Terzic [21]/2012	F/49	28	CAT, Valsalva maneuver	PM
Romeo [18]/2011	F/25	48	CAT	Tracheal compression
Maxwell [3]/2011	M/21	18 + 28 + 38 + 48	Inflating a balloon	PM
Hagr [14]/2010	F/69	48	Hydrogen peroxide	PM + infection/necrosis
Pousios [28]/2010	M/29	48	CAT	PM

F = female; M = male; NS = not specified; CAT = compressed air turbine; PM = pneumomediastinum; PT = pneumothorax; PR = pneumotach; PP = pneumopericardium.

**Table 2:** Management of 26 cases of subcutaneous emphysema after wisdom tooth extraction reported from January 2010 to April 2020.

First author (reference number)/year	AB	O_2_	Drainage	Intubation
Kaliszewski [22]/2020	+	−	−	−
North [1]/2019	+	−	+	+
Fehrle [25]/2019	+	−	−	−
Paschos [26]/2019	+	−	−	−
Tay [12]/2018	+	+	−	−
Zaheer [23]/2018	NS	NS	−	−
Jeong [24]/2018	+	−	−	−
	+	+	−	−
Thompson [8]/2017	+	−	−	−
Ocakcioglu [11]/2016	+	+	−	−
Picard [16]/2015	+	−	−	−
Tomasetti [4]/2015	+	−	−	−
Aslaner [10]/2015	+	+	+	−
Johannesma [20]/2014	+	−	−	−
Fleischman [13]/2014	+	−	+	−
Baisi [27]/2014	+	−	−	−
Lim [9]/2014	+	−	−	−
Kün-Darbois [17]/2014	NS	NS	−	−
Olate [2]/2013	+	−	−	−
Pilar [15]/2012	+	−	−	−
Chen [19]/2012	+	+	−	−
Terzic [21]/2012	+	−	−	−
Romeo [18]/2011	+	−	−	−
Maxwell [3]/2011	+	−	−	+
Hagr [14]/2010	+	−	+	+
Pousios [28]/2010	−	−	−	−

AB = antibiotics; O_2_ = oxygen therapy; NS = not specified; + = yes; − = no.

**Table 3:** Air distribution of the 26 cases of subcutaneous emphysema after wisdom tooth extraction reported from January 2010 to April 2020.

First author (reference number)/year	Periorbital	Facial	Cervical	Pneumomediastinum	Pneumopericardium	Pneumothorax
Kaliszewski [22]/2020	+	+	+	+	−	−
North [1]/2019	+	+	+	+	−	−
Fehrle [25]/2019	−	+	+	+	−	−
Paschos [26]/2019	−	+	+	+	−	+
Tay [12]/2018	−	+	+	+	−	−
Zaheer [23]/2018	−	−	+	+	−	−
Jeong [24]/2018	−	+	−	−	−	−
	−	+	+	+	−	−
Thompson [8]/2017	+	+	+	+	−	−
Ocakcioglu [11]/2016	−	−	+	+	−	+
Picard [16]/2015	−	+	+	+	−	−
Tomasetti [4]/2015	+	+	+	−	−	−
Aslaner [10]]/2015	−	+	+	+	−	−
Johannesma [20]/2014	−	+	+	+	+	−
Fleischman [13]/2014	+	+	−	−	−	−
Baisi [27]/2014	−	+	+	+	−	−
Lim [9]/2014	+	+	−	−	−	−
Kün-Darbois [17]/2014	−	+	+	+	−	−
Olate [2]/2013	+	+	+	−	−	−
Pilar [15]/2012	−	+	+	+	−	−
Chen [19]/2012	−	+	+	+	+	+
Terzic [21]/2012	−	+	+	+	−	−
Romeo [18]/2011	+	+	+	−	−	−
Maxwell [3]/2011	+	+	+	+	−	−
Hagr [14]/2010	−	+	+	+	−	−
Pousios [28]/2010	−	+	+	+	−	−

+ = yes; − = no.

**Table 4:** Causes of subcutaneous emphysema.

Latrogenic	Traumatic	Infectious	Spontaneous
Intubation	Perforating wounds	Necrotizing fasciitis	Alveolar rupture (chronic obstructive pulmonary disease, pulmonary emphysema)
Mechanical ventilation	Esophageal perforation (repeated vomiting)		Pneumothorax
Bronchoscopy	Pharynx/larynx/trachea injury		Postpartum healing
Tracheostomy	Barotrauma		
Head and/or neck surgery	Facial fractures		
Prolonged surgery			
Use of a continuous positive airway pressure machine			
Use of a peak flow meter			

**Table 5:** Incidence of emphysema symptoms and their distributions, as reported in the literature from January 2010 to April 2020.

	Data	Occurrences in the 26 cases reported in the literature, *n* (%)
Symptoms	Crepitations	25 (96%)
	Swelling	21 (80%)
	Dyspnea	9 (34%)
	Pyrexia	3 (11%)
	Epigastric pain	2 (7%)
	Chest pain	3 (11%)
	Neck pain	4 (15%)
	Facial pain	2 (7%)
	Dysphagia	5 (19%)
	Odynophagia	3 (11%)
	Diplopia	2 (7%)
Air distribution	Periorbital	9 (34%)
	Facial	24 (92%)
	Cervical	23(88%)
	Pneumomediastinum	20 (77%)
	Pneumothorax	3 (11%)
	Pneumopericardium	2 (7%)

## Data Availability

The data used to support the findings of this study are available from the corresponding author (Peters Mélissa) on reasonable request.
